# TET3 governs malignant behaviors and unfavorable prognosis of esophageal squamous cell carcinoma by activating the PI3K/AKT/GSK3β/β-catenin pathway

**DOI:** 10.1515/med-2022-0601

**Published:** 2022-12-02

**Authors:** Maoling Zhu, Bowen Shi, Chunguang Li, Shuchang Xu

**Affiliations:** Department of Gastroenterology, Yangpu Hospital, School of Medicine, Tongji University, Shanghai 200090, P.R. China; Department of Thoracic Surgery, Changhai Hospital, Navy Military Medical University, Shanghai 200438, China; Department of Thoracic Surgery, Shanghai Chest Hospital, Shanghai Jiao Tong University, Shanghai 200030, China; Department of Gastroenterology, Tongji Institute of Digestive Disease, Tongji Hospital, School of Medicine, Tongji University, Shanghai 200065, P.R. China

**Keywords:** ten–eleven translocation 3, esophageal squamous cell carcinoma, PI3K/AKT/GSK3β/β-catenin pathway, prognosis

## Abstract

Ten–eleven translocation 3 (TET3) participates in tumorigenesis and malignant transformation by mediating DNA demethylation and specific gene activation in malignances. This study aims to elucidate its molecular function and regulatory mechanism in esophageal squamous cell carcinoma (ESCC). Stable ESCC cells that infected with TET3 overexpression (OE) and knockdown lentiviral vector had been established. The biological behaviors and molecular mechanism of TET3 were demonstrated by cell biology experiments *in vitro* and *in vivo*. Tissues from patients with ESCC were used to demonstrate the clinical value of TET3. Our findings revealed that TET3 is highly expressed in ESCC tissues and related to poor prognosis of patients with ESCC. OE of TET3 presented a significant effect on proliferation, metastatic potential, and spheroid formation of ESCC cells by activating the PI3K/AKT/GSK3β/β-catenin axis. Knockdown of TET3 could remarkably reverse these malignant phenotypes. Patients with ESCC with high TET3 expression resulted in a shorter overall survival (OS) and disease-free survival. Based on the multivariate analysis, TET3 could be an independent favorable factor for predicting OS and recurrence. The high expression of TET3 not only aggravates malignant behaviors *in vitro* and *in vivo* but also becomes a novel biomarker for clinical monitoring and individualized precision treatment for patients with ESCC.

## 1 Introduction

Esophageal cancer (EC) is a malignant tumor derived from epithelial cells, in which incidence and mortality are seventh and sixth, respectively, in global human cancers [1]. Due to the insidious onset of EC, a considerable number of patients with EC have developed to advanced stages at their first diagnosis. Postoperative metastasis and recurrence are the key factors seriously restricting the prognosis and long-term survival of patients with EC [2,3,4,5]. Histologically, EC can be divided into esophageal squamous cell carcinoma (ESCC) and esophageal adenocarcinoma, among which ESCC is currently the main histological type in China and western countries [6]. Up to now, surgery is still the first choice for clinical radical treatment of ESCC in combination with adjuvant therapy [7,8]. Despite the continuous improvement in the clinical early diagnosis and combined treatment of EC, the prognosis is still far from satisfactorily [9]. Elucidating the underlying mechanism will undoubtedly benefits the clinical prevention and targeted therapies of ESCC.

The formation, evolution, Infiltration and metastasis of ESCC are complex biological processes involving multi-molecular participation and mutual regulation, among which epigenetic variation and modification might play a crucial role [10]. Ten–eleven translocation (TET) family members are newly identified DNA demethylases including TET1, TET2, and TET3. The main biological function of TETs is converting 5-methylcytosine to generate 5-hydroxymethylcytosine, thereby mediating DNA demethylation and specific gene activation [11]. Dysregulation of TETs in several malignant tumors has been reported, including breast cancer [12], hepatocellular carcinoma [13], melanoma [14], glioma [15], renal cell carcinoma [16], and colorectal cancer [17]. Although accumulating evidence has shown that TETs are associated with tumorigenesis and progression, the specific mechanism remains highly mysterious.

In this present study, we confirmed that TET3 was overexpressed in ESCC. Further studies identified that PI3K/AKT/GSK3β/β-catenin pathway drives ESCC through TET3 regulation for stem cell-like maintenance and survival. TET3 has the potential to become a new biomarker or a therapeutic target for ESCC.

## 2 Materials and methods

### 2.1 Patients and collection of tissue samples

ESCC tissue samples and paired para-cancerous tissue samples (*n* = 62) were enrolled from patients, which were pathologically diagnosed as primary ESCC and received surgical resection from March 2013 to January 2014 at Changhai Hospital, Navy Military Medical University (Shanghai, China). All of the patients did not receive preoperative adjuvant therapy including radiotherapy, chemotherapy, or biotherapy. The extraction of total RNA from ESCC tissues was carried out by conventional steps and detected by using the quantitative real-time PCR (RT-qPCR) assay. The extraction of total protein from 7 pairs of ESCC tissues was conducted and detected by using the western blot assay. All clinical samples were pathologically confirmed by two independent pathologists and was followed up until January 2019.

### 2.2 Immunohistochemistry (IHC)

We adopted IHC to evaluate the protein expression of TET3 in ESCC tissues. The embedded tissues were cut into 4-µm-thick serial sections. The information of antibodies is listed as follows: anti-TET3 antibody (dilution, 1:300; ab153724; Abcam; UK), anti-EpCAM antibody (dilution, 1:500; ab221552; Abcam), anti-β-catenin antibody (dilution, 1:500; ab32572; Abcam), anti-p-AKT (T308) antibody (dilution, 1:100; ab38449; Abcam), anti-p-AKT (S473) antibody (dilution, 1:100; ab81283; Abcam), anti-p-GSK3β (Tyr216 + Tyr279) antibody (dilution, 1:200; ab68476; Abcam), anti-p-GSK3β (Ser9) antibody (dilution, 1:200; ab75814; Abcam), anti-GSK3β antibody (dilution, 1:5,000; ab32391; Abcam), and anti-AKT antibody (dilution, 1:1,000; ab8805; Abcam). We replaced the primary antibody with phosphate-buffered solution as the negative control. The results were reviewed and evaluated by two experienced pathologists. The number and proportion of positive cells was evaluated by analyzing 10 randomly fields. And the immunoreactive scoring was performed based on a quick scoring system. According to the intensity of protein staining, we divided all samples into four grades: 0 indicated no staining, 1 indicated weak staining, 2 indicated intermediate staining, and 3 indicated strong staining. Percentage scores were evaluated as 0 (0%), 1 (1–25%), 2 (25–50%), 3 (50–75%), and 4 (75–100%). We multiply the staining intensity grade by the percentage score, and the median value of total scores was identified as the optimal cutoff value. A score of >4 was considered as high expression.

### 2.3 Cell culture

Human normal esophageal epithelial cell line (HEEC) was used as normal cell. ESCC cell lines Eca109 and TE1 were obtained from Shanghai Cell Bank of Chinese Academy of Sciences (Shanghai, China), which were conventionally cultured in RPMI 1640 (GIBCO, Grand Island, NY, USA) supplemented with 10% fetal bovine serum (GIBCO, USA) at 37°C in 5% CO_2_.

### 2.4 Spheroid formation assay

For spheroid formation, ESCC cells with different treatment groups were digested into suspensions and then seeded in ultra-low adhesion cell plates (Corning, USA) with complete medium (1 × 10^3^ cells/well). The number of spheroid clusters was counted every other day until day 7.

### 2.5 Overexpression (OE) and knockdown of TET3

For TET3 OE, lentiviral vector (pLV-EF1α-EGFP-N plasmid; Inovogen, VL3311) containing full-length human TET3 cDNA (NM_001287491.2) and puromycin resistance gene was imported in 293T cells using cloned or purchased vectors. The shRNA1 and shRNA2 were designed and constructed into pLKO.1 plasmids (Addgene, Plasmid #10878) as TET3 knockdown vectors. The non-targeting RNA (ntRNA, pLKO) (GCGCGATAGCGCTAATAATTT), shRNA1 (GAACCTTCTCTTGCGCTATTT) (KD1), and shRNA2 (ACTCCTACCACTCCTACTATG) (KD2) sequences were cloned into vector, and Eca109 or TE1 cells were infected with shRNA or TET3-OE lentiviruses (MOI = 20). The cell sublines with OE or KD of TET3 were confirmed by the western blot assay.

### 2.6 RT-qPCR

The extraction of total RNA from cells and tissue samples was conducted by using TRIzol reagent (Invitrogen, USA) conventionally. We evaluated the quality and concentration of extracted RNA by detecting the absorbance of RNA solution (OD260/OD280). RT-qPCR was carried out by using a SYBR Premix Ex Taq™ II kit (Takara, China). Expression value of target gene was calculated automatically using 2^−ΔΔCt^. PCR primers for TET3 and GAPDH were synthesized (Sangon, China) as follows: TET3: forward 5′-GTTCCTGGAGCATGTACTTC-3′ and reverse 5′-CTTCCTCTTTGGGATTGTCC-3′; GAPDH: forward 5′-CACCCACTCCTCCACCTTTG-3′ and reverse 5′-CCACCACCCTGTTGCTGTAG-3′.

### 2.7 Western blot analysis

The extraction of total protein from cells and tissue samples was performed by radio-imunoprecipitation assay lysis buffer with 1% phenylmethanesulfonyl fluoride, 1% phosphatase inhibitor, and 1% protease inhibitor (Beyotime Biotech, China). The experimental procedure was carried out as usual. The information of antibodies is listed as follows: anti-TET3 antibody (dilution, 1:1,000; ab153724; Abcam), anti-β-catenin antibody (dilution, 1:2,000; ab32572; Abcam), anti-p-AKT (T308) antibody (dilution, 1:1,000; ab38449; Abcam), anti-p-AKT (S473) antibody (dilution, 1:1,000; ab81283; Abcam), anti-p-GSK3β (Tyr216 + Tyr279) antibody (dilution, 1:2,000; ab68476; Abcam), anti-p-GSK3β (Ser9) antibody (dilution, 1:2,000; ab75814; Abcam), anti-GSK3β antibody (dilution, 1:5,000; ab32391; Abcam), anti-AKT antibody (dilution, 1:1,000; ab8805; Abcam), and anti-GAPDH antibody (dilution, 1:5,000; ab9482; Abcam).

### 2.8 Colony formation assay

ESCC cells (1 × 10^3^ cells/well) were seeded into six-well culture plates with complete medium. Cell colonies were fixed with 4% formaldehyde and stained with crystal violet staining solution (Beyotime Biotech) after culturing for 2 weeks. The number of clones was counted by using light microscope. Three independent experiments in each group were carried out.

### 2.9 Cell migration and invasion assay

The experimental procedure was conducted conventionally. In wound-healing assays, ImageJ software (V1.8.0, NIH, USA) was adopted to measure the injury aera. In Transwell assay, cells that passed through upper chambers were fixed using methyl alcohol and stained with crystal violet staining solution (Beyotime Biotech). Three independent experiments in each group were carried out.

### 2.10 Cellular immunofluorescence

The cell spheroids were collected after 7 days culture and identified by cellular immunofluorescence detection. The experimental procedure was carried out as usual. The information of antibodies is listed as follows: anti-EpCAM antibody (dilution, 1:100; ab221552; Abcam), anti-β-catenin antibody (dilution, 1:250; ab32572; Abcam), and anti-CD133 antibody (dilution, 1:200; D2V8Q; CST, USA).

### 2.11 Animal experiment

Animal experiments have been complied with the permission approved by the Committee on Ethics of Medicine, Navy Military Medical University. All animals were raised under SPF condition, all experimental procedures were in accordance with the criteria. Twenty-six-week-old BALB/c male nude mice were randomly divided into four groups (five mice/each group): control group (EGFP), TET3-OE group (OE), control group (pLKO), and TET3-knockdown group (KD1). In subcutaneous model, ESCC cells (1 × 10^7^ cells/mouse) were subcutaneously injected into each mouse and raised for 42 days. After that, mice were sacrificed painlessly and tumor volume and weight were calculated. In the lung metastasis model, ESCC cells (5 × 10^5^ cells/mouse) were injected into the tail vein and raised for 30 days. Mice were sacrificed and dissected to assess the number of metastatic nodules in lungs.

### 2.12 Statistical analysis

GraphPad Prism 5.0 software and SPSS 22.0 software were used for statistical analysis. Data were presented as mean ± standard deviation. Statistical differences between groups were tested by using Student’s *t*-test or one-way analysis of variance test. Kaplan–Meier survival method was used to analyze survival condition. *P* < 0.05 indicated that the difference was statistically significant.


**Ethics statement:** The collection and use of clinical resources, including ESCC tissue samples and follow-up data, and *in vivo* animal models, which performed in accordance with guidelines, was complied with the permission of Biomedical Ethics Committee of Navy Military Medical University (Approval Number: CHEC2020-021; date: 06/02/2020; Shanghai, China). The written consent from each patient has been obtained in accordance with the Declaration of Helsinki.

## 3 Results

### 3.1 TET3 is overexpressed in ESCC

**Figure A1:**
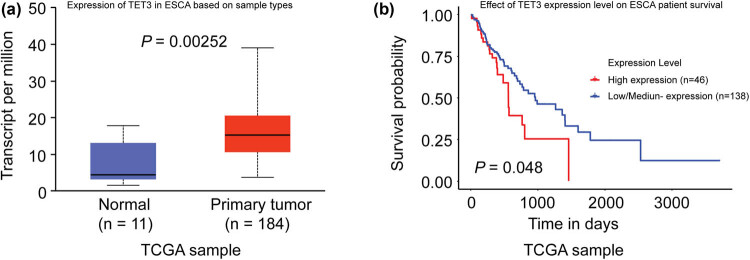
Overexpression and knockdown of TET3 in ESCC cells. (a) Fluorescence microscopy confirmed that TET3 was located in cytoplasm and nuclei (scale bar = 50 μm) at 72 h after infection with lentiviral vectors (MOI = 20). (b) Western blot assay was used to detect the protein level of TET3 in OE and KD cell sublines.

Based on the data from UALCAN (Data from TCGA database) (http://ualcan.path.uab.edu), we compared the mRNA expression of TET3 between ESCA tissues (*n* = 184) and normal tissues (*n* = 11). The results revealed that the expression of TET3 was elevated in tumors than that in normal tissues (*P* = 0.00252) (Figure A1(a)). We further focus on the correlation between the mRNA levels of TET3 and the survival time of patients with ESCA by using publicly available datasets (UALCAN). The results indicated that patients with ESCA with the high expression of TET3 mRNA were correlated with shorter survival time (*P* = 0.048) (Figure A1(b)).

**Figure 1:**
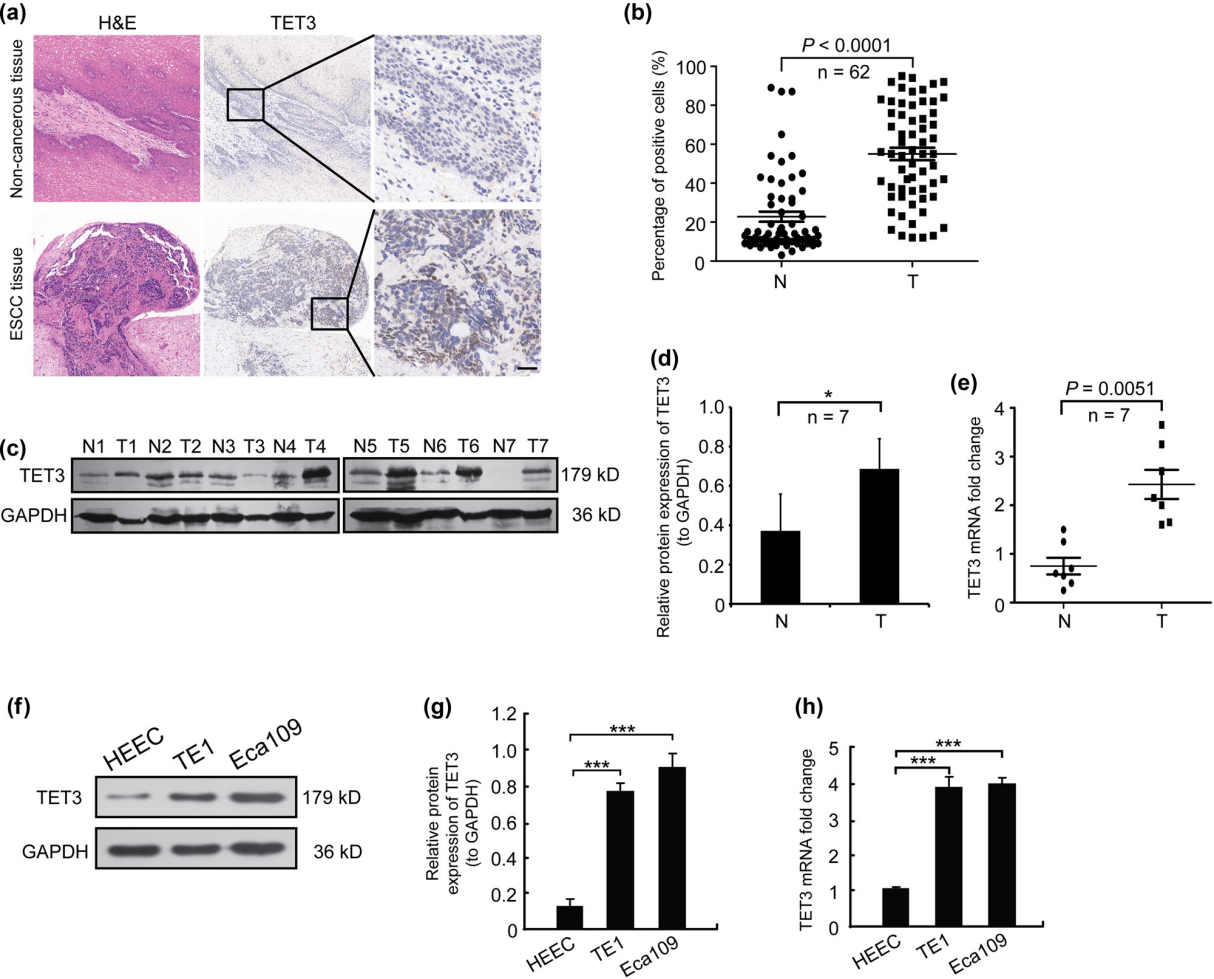
TET3 is up-regulated in ESCC. (a) Representative fields of TET3 protein level in ESCC tissue samples (scale bar = 50 μm). (b) Statistical analysis of the proportion of positive cells in immunohistochemical results of ESCC tissue sections. (c) Protein levels of TET3 in paired ESCC tissues (*n* = 7). (d) Statistical analysis of the protein levels of TET3 in paired ESCC samples (*n* = 7). (e) RT-qPCR assays were used to measure TET3 mRNA in ESCC tissues. (f) Protein levels of TET3 in ESCC cell lines. (g) Statistical analysis of the protein expression levels of TET3 in ESCC cell lines. (h) RT-qPCR assays were used to detect TET3 mRNA in ESCC tissues. *P < 0.05, ***P < 0.001. N, non-tumor tissue; T, tumor tissue.

To confirm the above conclusion, we detected the protein expression of TET3 in 62 ESCC samples and para-cancerous tissues by using IHC. The results suggested that TET3 was located in cytoplasm and nuclei, which were elevated in ESCC tissues compared with paired para-cancerous tissues (Figure 1a and b). We collected seven pairs of freshly surgically resected ESCC tissues and extracted total RNA and protein using the RT-qPCR assay and the western blot analysis, respectively. Our results revealed that the protein and mRNA levels of TET3 were dramatically up-regulated in ESCC tissues (Figure 1c–e). Meanwhile, the protein and mRNA expression of TET3 in ESCC cell lines (TE1 and Eca109) were up-regulated compared with normal HEEC (Figure 1f–h). According to these results, we confirmed that TET3 was abnormally up-regulated in ESCC.

### 3.2 TET3 promotes the proliferation and metastasis of ESCC cell lines *in vitro* and *in vivo*

**Figure A2:**
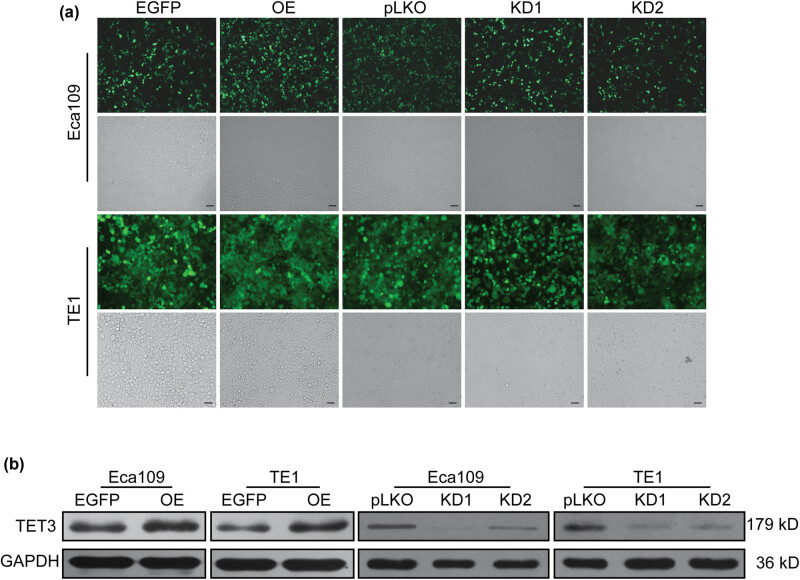
TET3 is over-expressed in ESCA and is correlated to poor prognosis and survival. (a) TET3 mRNA is up-regulated in ESCA samples (TCGA data). (b) Survival analysis suggested that elevated TET3 resulting in shorter overall survival.

To explore the biological function of TET3, endogenous OE and knockdown cell sublines were established in TE1 and Eca109. The exogenous TET3-EGFP was stably expressed in TE1 (OE) and Eca109 (OE). The transcription level of TET3 was stably down-regulated by two different shRNA vectors in TE1 (KD) and Eca109 (KD). Fluorescence microscopy showed that TET3 was located in cytoplasm and nuclei (Figure A2(a)). The OE and knockdown level of TET3 in ESCC cells were confirmed by the western blot analysis (Figure A2(b)).

**Figure 2:**
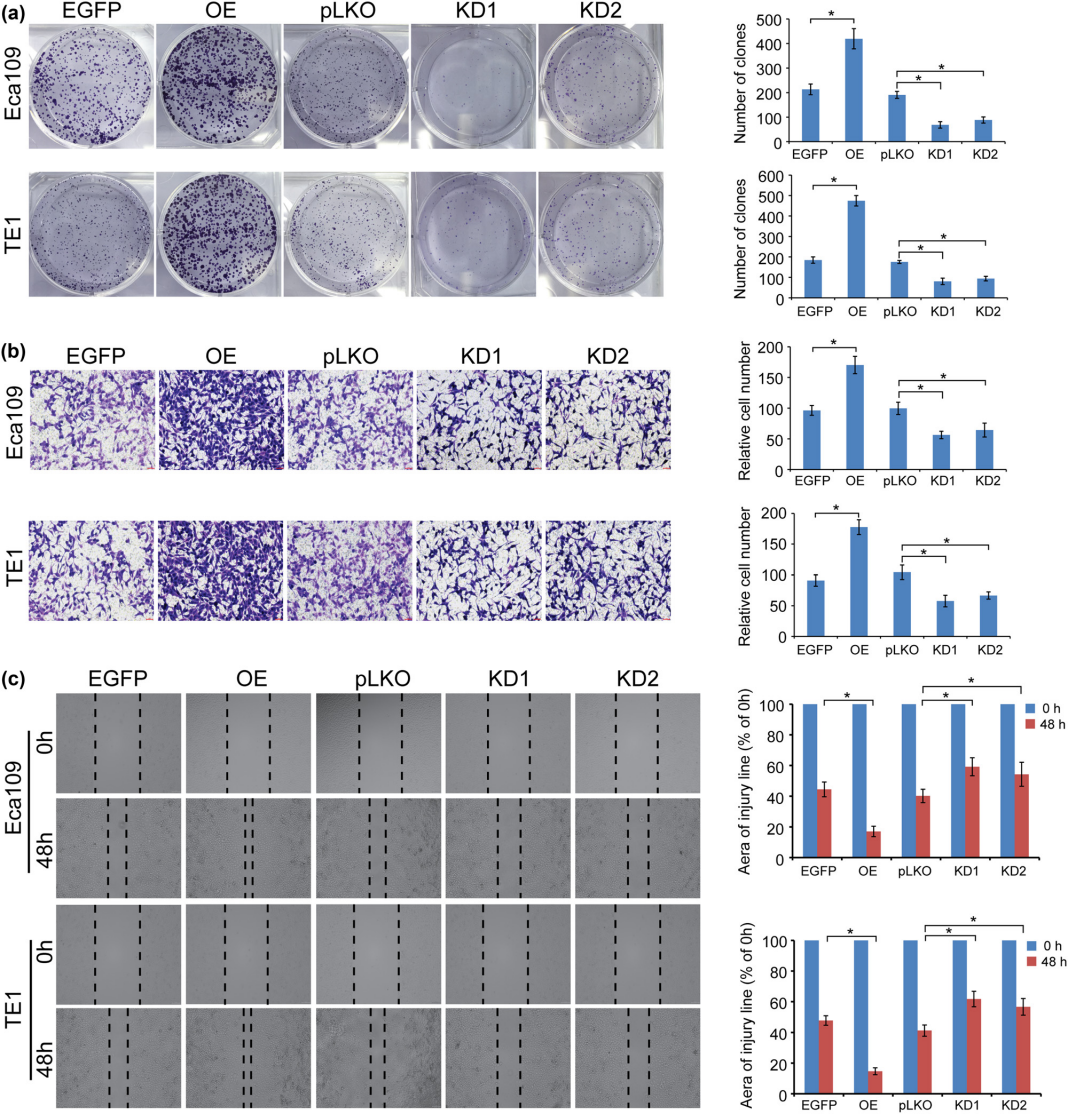
High expression of TET3 promotes the viability and motility of ESCC cells *in vitro*. (a) TET3 increased the colony formation ability of ESCC cells. (b) TET3 elevates the invasion abilities of ESCC cells that were evaluated by Transwell chamber assays. (c) Cell wound-healing assays were conducted to measure the migration potential of ESCC cells. *P < 0.05.

Colony formation, Transwell, and wound-healing assays were employed to evaluate the proliferation and motility of TET3-OE/KD Eca109 and TE1 cells. Our results found that the OE of TET3 significantly promoted the ability of proliferation and the motility of ESCC cells, while knockdown of TET3 exhibited the opposite outcomes (Figure 2a–c).

**Figure 3:**
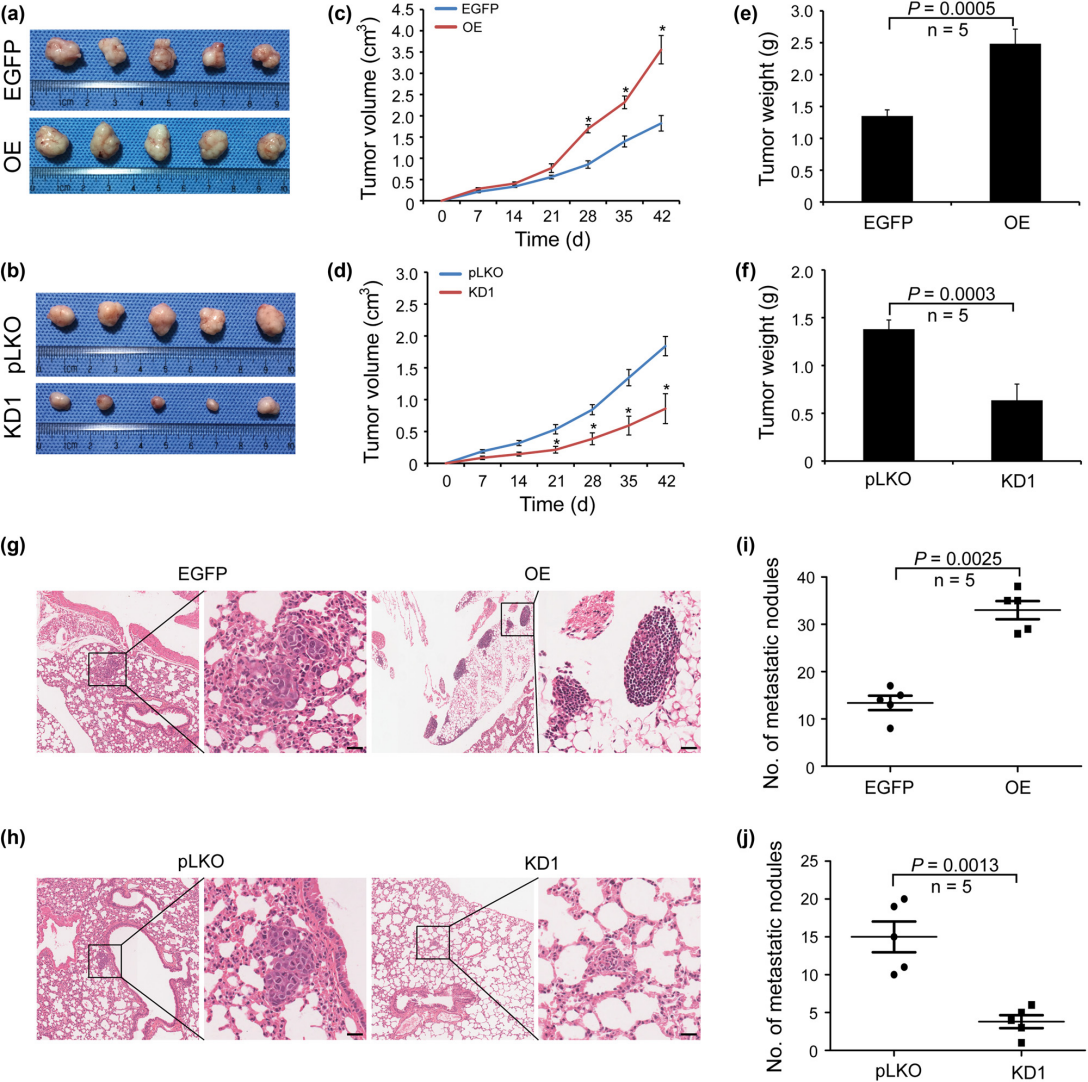
TET3 promotes proliferation and metastasis of ESCC cells *in vivo*. (a–f) Eca109 treated with OE and knockdown of TET3 subcutaneously injected in nude mice to construct animal model. Tumor volumes and weights were measured. *n* = 5 mice per each group. (g–j) Representative fields of metastatic tumor nodules in lung of each group of nude mice (scale bar = 50 μm). The numbers of metastatic tumor nodules in lung were statistically analyzed. Five mice per each group. *P < 0.05.

Animal models by subcutaneous and tail vein injection in nude mice were established to evaluate the function of TET3 on tumorigenicity and metastatic potential of ESCC cells *in vivo*. The results revealed that the tumor volume and weight in TET3-OE group were significantly larger than those in the control group (Figure 3a–c). By contrary, the tumor volume and weight in TET3-KD group were significantly decreased compared with those in the control group (Figure 3d–f). HE staining showed that the number of metastatic nodules in lung tissues of TET3-OE group was statistically more than that in the control group, and TET3-KD group had the least number of lung metastatic nodules (Figure 3g–j). Our present results suggested that the high expression of TET3 could promote the proliferation and metastasis potential of ESCC cells *in vitro* and *in vivo*.

### 3.3 TET3 promotes spheroid formation of ESCC cells *in vitro*

**Figure 4:**
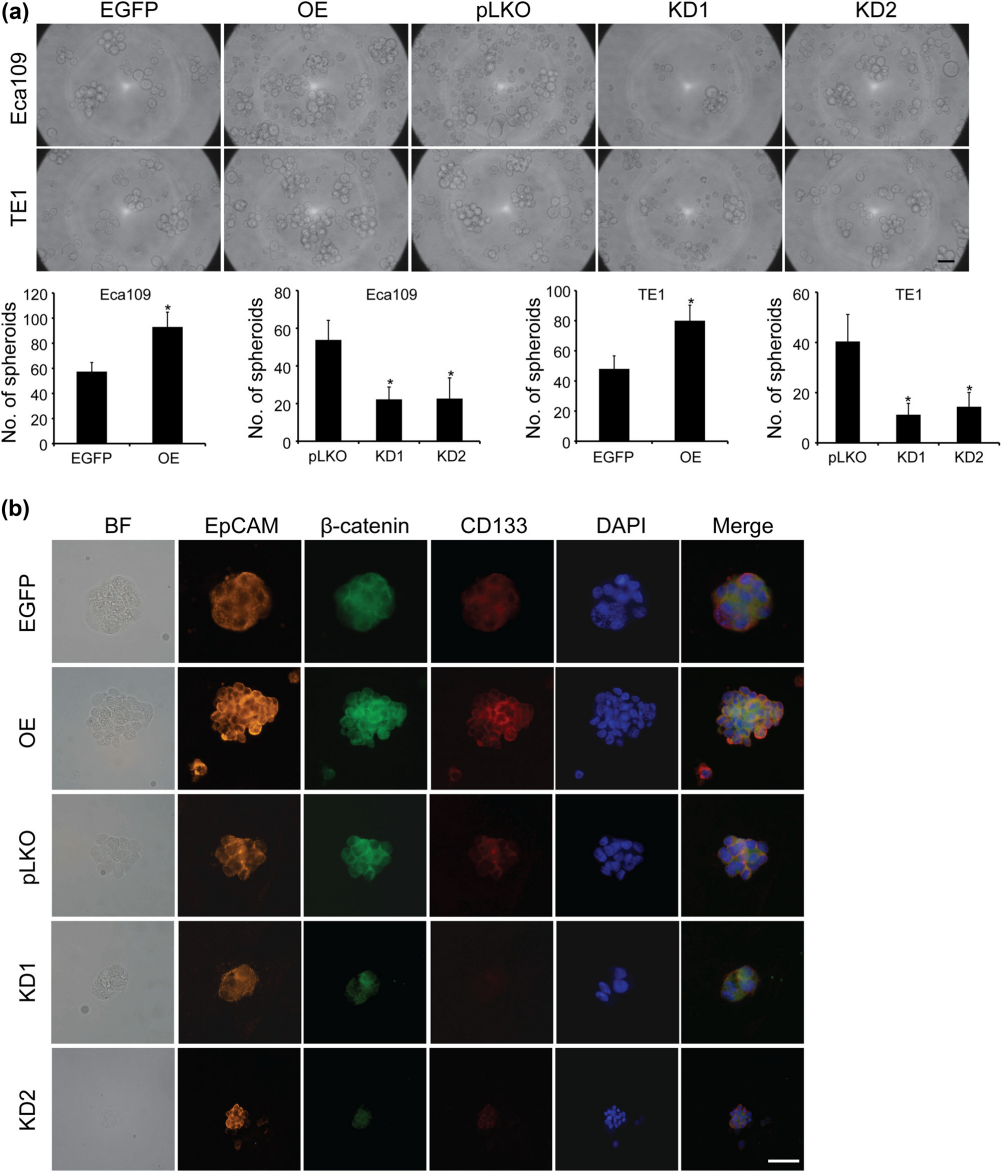
TET3 promotes ESCC cell spheroid formation. (a) Three-dimensional spheroid assay was conducted to measure the spheroidizing ability of ESCC cells. (b) Immunofluorescence of stem cell markers, including EpCAM, β-catenin, and CD133, in Eca109, scale bar = 50 μm, *P < 0.05 vs control group.

To further explore the effect of TET3 on the maintenance of stem cell-like characteristics in ESCC cells, we examined the expression of candidate cancer stem cell (CSC) markers. Three-dimensional spheroid formation assay was used to evaluate the spheroidizing potential of ESCC cells. In the TET3-OE group, the ability of tumor cells to form spheroids was increased. Down-regulation of TET3 inhibited the formation of spheroids (Figure 4a). The stem cell markers in ESCC cell spheroids were measured by immunofluorescence staining, and we found that the stem cell markers, including EpCAM, β-catenin, and CD133 were positive (Figure 4b). While knockdown of TET3 down-regulated the protein level of β-catenin and CD133 in spheroids. These results demonstrated that the spheroids have the characteristics of stem cells (Figure 4b). Down-regulation of TET3 could inhibit the formation of ESCC cell spheroids and decreased the expression level of CSC markers. In summary, high expression of TET3 could promote the formation of spheroids and the maintenance of stem cell-like characteristics in ESCC cells.

### 3.4 TET3 enhances the malignant transformation of ESCC cells by activating the PI3K/AKT/GSK3β/β-catenin axis

It has been widely recognized that PI3K/AKT signaling pathway could participate in the malignant progression of a variety of human tumors by regulating the growth of tumor cells, invasion, metastasis, and angiogenesis and maintaining the characteristics of tumor stem cells [18,19]. GSK3β is a crucial substrate in the PI3K/AKT signaling pathway and regulates several physiological processes, such as the activity of mitochondria [20].

**Figure 5:**
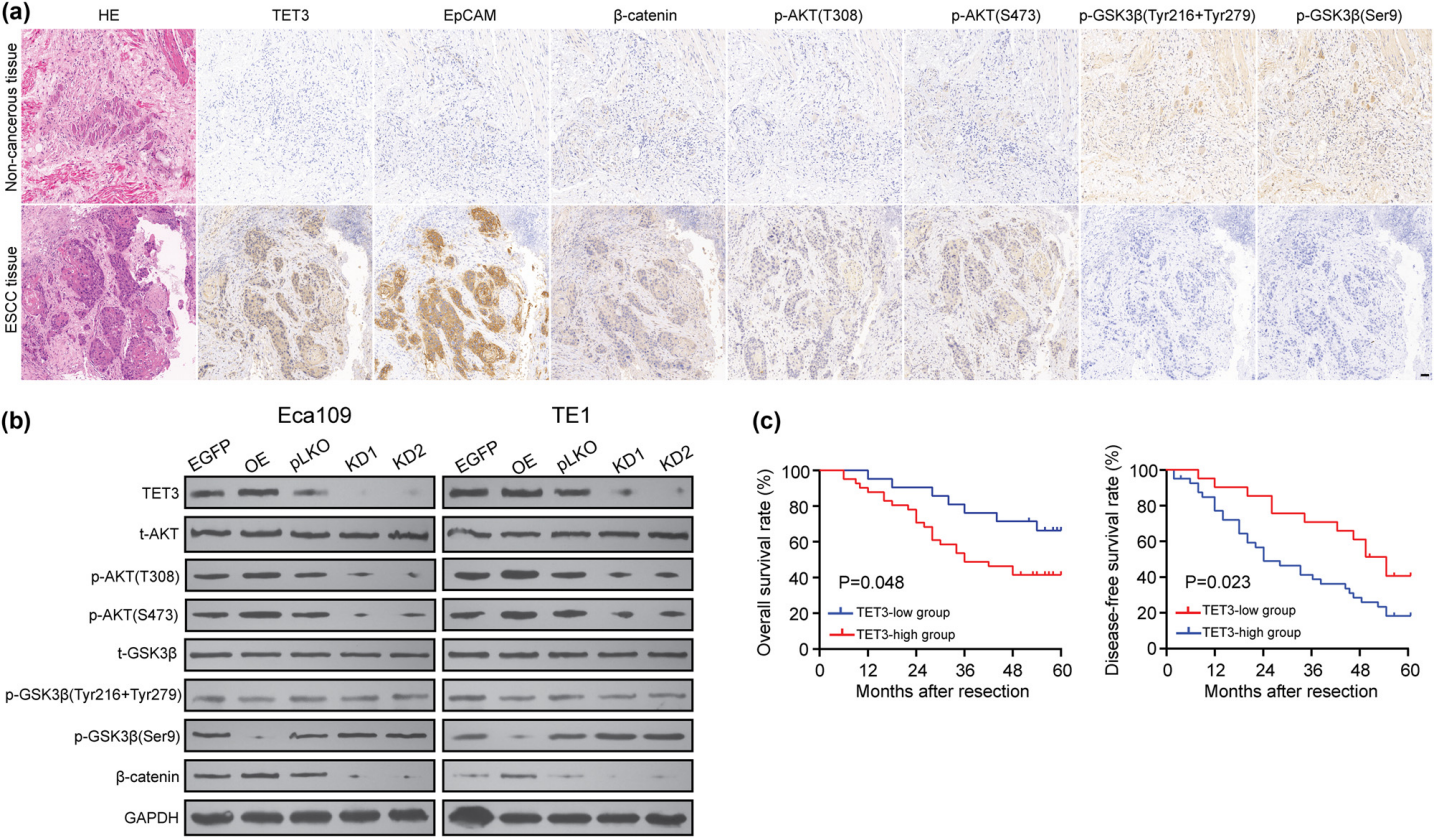
TET3 increased the viability, motility, and stem cell characteristics of ESCC cells by regulating the PI3K/AKT/GSK3β/β-catenin axis. (a) Representative fields of signaling pathway-related protein expression, including TET3, EpCAM, β-catenin, p-AKT (T308 and S473), and p-GSK3β (Tyr216/279 and Ser9), in ESCC samples (scale bar = 50 μm). (b) Western blot assay was used to detect the expression of signaling pathway-related protein in ESCC cells. (c) OS and DFS analyses of TET3 in patients with ESCC (*n* = 62).

Based on IHC results of serial tissue sections of ESCC, the protein level of p-AKT (T308), p-AKT (S473), EpCAM, and β-catenin was up-regulated; meanwhile, p-GSK3β (Tyr216 + Tyr279) and p-GSK3β (Ser9) are relatively low (Figure 5a). In addition, the up-regulation of TET3 led to a significant increase in p-AKT (T308), p-AKT (S473), and β-catenin while inhibited the expression of p-GSK3β (Tyr216 + Tyr279) and p-GSK3β (Ser9) in ESCC cells. Knockdown of TET3 expression led to the activation of the expression of p-GSK3β (Ser9); meanwhile, it down-regulated p-AKT (T308), p-AKT (S473), and β-catenin, but the expression changes in p-GSK3β (Tyr216 + Tyr279) were not obvious (Figure 5b). According to these results, we could infer that TET3 inhibits the phosphorylation level of GSK3β (especially on Ser9 site) through the activated PI3K/AKT signaling pathway, enhancing the stability of β-catenin in cancer cells and subsequently participating in downstream gene transcription related to cell proliferation and metastasis.

### 3.5 High expression of TET3 leads to poor prognosis for patients with ESCC

The expression pattern of TET3 and clinicopathological indicators of patients with ESCC was analyzed. These results indicated that the gender, age, degree of differentiation, and TMN staging were not correlated with the OE of TET3 of patients with ESCC (Table 1). The elevated expression of TET3 was significantly correlated with lymph node metastasis (*P* = 0.047). According to the staining scores of TET3, we divided 62 patients with ESCC into the high expression group (*n* = 41) and the low expression group (*n* = 21). The patients were followed up from 6 months to 60 months, and the median time was 42.2 ± 18.1 months. Kaplan–Meier survival analysis was carried out to analyze the expression of TET3 on the clinical prognosis of patients with ESCC. As presented in Figure 5c, high expression of TET3 was related to lower overall survival (OS) rate (*P* = 0.048) and disease-free survival (DFS) rate (*P* = 0.023) compared with the TET3-low group. Obviously, OE of TET3 was significantly correlated to the poor prognosis and survival of patients with ESCC. Based on the univariate analysis, we selected lymph node metastasis (*P* = 0.001), TMN staging (*P* = 0.003), and TET3 expression (*P* = 0.001) for multivariate analysis. The results revealed that TET3 has the potential to be an independent favorable factor for OS and recurrence (*P* = 0.001, Table 2). Collectively, clinical analysis suggested that elevated expression of TET3 may promote the malignant transformation of ESCC and is closely related to poor prognosis.

**Table 1:** Correlation between TET3 expression and ESCC clinicopathological features

Variables	*N* (*N* = 62)	TET3 High	TET3 Low	*χ* ^2^	*P*
Gender		41	21	0.014	0.907
Male	39	26	13		
Female	23	15	8		
Age (years)				0.192	0.661
≤60	26	18	8		
>60	36	23	13		
Differentiation				2. 254	0.324
Grade 1	19	10	9		
Grade 2	33	24	9		
Grade 3	10	7	3		
Lymphatic metastasis				3.961	0.047
No	22	11	11		
Yes	40	30	10		
TNM staging				3.734	0. 053
I–II	25	13	12		
III–IV	37	28	9		

TET3: ten–eleven translocation 3; ESCC: esophageal squamous cell carcinoma; TNM: tumor, lymph node, and metastasis.

**Table 2:** Univariate and multivariate Cox’s proportional hazard models (*n* = 62)

Term	Risk ratio	95% confidence interval	*P* value
**Univariate**			
Gender	1.082	0.915–1.279	0.358
Age	0.924	0.781–1.094	0.360
Differentiation	1.110	0.980–1.257	0.100
Lymphatic metastasis	1.466	1.230–1.747	0.001
TNM staging	1.292	1.093–1.527	0.003
TET3	2.225	1.860–2.662	0.001
**Multivariate**			
Lymphatic metastasis	1.128	0.965–1.318	0.130
TNM staging	1.059	0.915–1.227	0.442
TET3	1.953	1.664–2.292	0.001

TET3: ten–eleven translocation 3; ESCC: esophageal squamous cell carcinoma; TNM: tumor, lymph node, and metastasis.

## 4 Discussion

In our present study, we found that TET3 was abnormally overexpressed in ESCC cells and tissues, which could be acted as an oncogene by facilitating malignant progression and predicting poor prognosis. Tumor recurrence and metastasis are multi-step processes, which usually accompanied by specific genomic, proteomic, metabolic, and epigenetic changes [21,22,23]. In recent decades, accumulating novel biomarkers could be used for early diagnosis and clinical therapy, accompanied with studies focus on the mechanism of tumorigenesis, and progression has made remarkable achievements. However, it is regrettable that some clinical challenges, such as the precise molecular mechanism of tumorigenesis, proliferation, and metastasis, maintenance of tumor stem-like characteristics, acquired drug resistance including chemotherapeutic drugs, and radiotherapy, still have not been well solved.

ESCC is one of the death-related tumors in China [24]. Postoperative recurrence and metastasis of ESCC are the crucial factors restricting the long-term survival of patients [25]. Based on the sequencing data by our group, screening and analyzing the public databases (http://ualcan.path.uab.edu), we locked TET3 as the target molecular. We investigated the function and possible mechanism of TET3 in ESCC, for the purpose of elucidating the molecular mechanism, regulatory function, and the clinical value in ESCC.

As we know, TET family members were dysregulated in various tumors, which play the function of oncogene or tumor suppressor gene. TET3 was decreased in ovarian cancer and silencing of which could inhibit malignant transformation of cancer cells [26]. But in acute myeloid leukemia, TET3 expression was significantly increased and could be used as an independent prognostic factor [27]. Therefore, the biological function of TET3 may be tissue specific. Combined with the information of the public database and our present results, we validated that TET3 was dramatically overexpressed in ESCC, which was consistent with Murata’s experimental results [28]. That is to say, TET3 could serve as an oncogene in the initiation and progression of ESCC. Furthermore, molecular mechanism study found that up-regulation of TET3 could elevate the proliferation and metastasis ability of ESCC cells. This phenomenon has also been verified in nude mice. Deprivation of TET3 expression remarkably inhibited the proliferation and metastatic ability of ESCC cells *in vitro* and *in vivo*. Meanwhile, based on the clinical data, we got a consistent result that elevated level of TET3 was significantly correlated with poor prognosis of patients with ESCC.

Studies have shown that OE of TET3 contributes to inducing and maintaining the CSC-like phenotype [29,30,31]. Recent findings revealed that, as a functional approach, spheroid formation is particularly useful to enrich the potential CSC subpopulations [32,33]. In this study, we found that OE of TET3 could enhance the spheroidization and the expression level of CD133 and β-catenin in ESCC cells *in vitro*. Down-regulation of TET3 decreased the ability of spheroid formation, meanwhile the expression of above stem cell-related markers was also significantly inhibited. These CSC-like cells may have significant growth advantages and the ability to resist survival pressure, even led to tumor proliferation and metastasis under specific physiological condition.

It has been widely recognized that PI3K/AKT is a classic signaling pathway, which participates in malignant progression of a variety of human malignances by regulating proliferation of tumor cells, invasion and metastatic potential, angiogenesis and maintaining the characteristics of tumor stem cells [18,19]. GSK3β is a crucial substrate in the PI3K/AKT signaling pathway and regulates the activity of mitochondria [20]. Abnormal phosphorylation level of GSK3β could affect Wnt/β-catenin/T-cytokine (TCF) signals and mediate epithelial mesenchymal transformation (EMT) process by participating in the transcriptional regulation of snail (EMT-related transcription factor) expression [34,35]. However, activation of PI3K/AKT signaling pathway could suppress GSK3β phosphorylation at the Ser9 site, which enhances the stability of β-catenin in cells, promotes its nuclear translocation, and eventually participates in the transcriptional regulation of downstream cell proliferation and metastasis-related genes [20,36]. Our present study revealed that the expression of TET3 was significantly positively correlated with β-catenin and the phosphorylation level of AKT (T308 and S473) was negatively correlated with the phosphorylation level of GSK3β (Ser9). These results confirmed that TET3 could inhibit the phosphorylation level of GSK3β (mainly through Ser9 site) by activating the AKT signaling pathway, eventually to stabilize and promote the β-catenin expression in ESCC cells.

Taken together, our data suggested that TET3 may act as an oncogene in ESCC. Elevated expression of TET3 could significantly facilitate the malignant progression, including proliferation, metastasis, and maintenance of stem cell characteristics in ESCC cells via PI3K/AKT/GSK3β/β-catenin pathway. The importance of TET3 in prognostic survival has also been validated in patients with ESCC. TET3 is expected to become a novel molecular marker for clinical diagnosis, treatment, and monitoring prognosis in the future.

## Abbreviations

TET3: ten–eleven translocation 3
ESCC: esophageal squamous cell carcinoma
IF: immunofluorescence
IHC: immunohistochemistry
qRT-PCR: quantitative real-time PCR
KD: knockdown
OE: overexpression
N: non‑tumor tissue
T: tumor tissue
CSC: cancer stem cell
TNM: tumor, lymph node, and metastasis
EpCAM: epithelial cell adhesion molecule
OS: overall survival
DFS: disease-free survival.
